# The role of gut dysbiosis in endocrine and metabolic derangements of chronic kidney disease: mechanisms, controversies, and future perspectives

**DOI:** 10.3389/fendo.2026.1854704

**Published:** 2026-05-29

**Authors:** Zhiqiang Ouyang, Wanjun Hu, Fangyu Zhu, Yafei Zhang, Lijuan Wang, Qiuwen Ye

**Affiliations:** 1 Department of Radiology, Yan’an Hospital of Kunming City (Yan’an Hospital Affiliated to Kunming Medical University), Kunming, China; 2 Key Laboratory of Cardiovascular Disease of Yunnan Province, Kunming, China; 3 Key Laboratory of Tumor Immunological Prevention and Treatment of Yunnan Province, Kunming, China; 4 Department of Nuclear Magnetic Resonance, The Second Hospital & Clinical Medical School, Lanzhou University, Lanzhou, China; 5 Department of Urology, The First People’s Hospital of Kunming, Kunming, China; 6 Hepatobiliary and Pancreatic Surgery, Yunnan Cancer Hospital (The Third Affiliated Hospital of Kunming Medical University), Kunming, China

**Keywords:** chronic kidney disease, chronic kidney disease–mineral and bone disorder, endocrine-metabolic derangements, gut dysbiosis, gut–kidney axis, uremic toxins

## Abstract

Chronic kidney disease (CKD) is increasingly recognized as a systemic endocrine–metabolic disorder in which gut dysbiosis acts as a key amplifier of disease progression and complications. This mini-review summarizes current evidence linking alterations in gut microbial composition and function to major endocrine and metabolic derangements in CKD. Dysbiosis promotes the accumulation of gut-derived uremic toxins, including indoxyl sulphate, p-cresyl sulphate, and trimethylamine N-oxide, while reducing beneficial metabolites such as short-chain fatty acids, vitamin K, and protective tryptophan derivatives. Through interconnected pathways involving inflammation, oxidative stress, endothelial injury, immune dysregulation, and disturbed inter-organ signalling, these changes contribute to chronic kidney disease–mineral and bone disorder, vascular calcification, insulin resistance, protein–energy wasting, anaemia, and cognitive dysfunction. We further discuss major controversies, including the context-dependent role of trimethylamine N-oxide, the contested operational definition of “dysbiosis,” and the clinical balance between low-protein dietary strategies and the risk of malnutrition, as well as the role of dietary fibre and whole-diet patterns. Practical diet-related approaches that translate these mechanistic findings into clinical practice are also outlined. Current limitations include incomplete mechanistic validation, limited longitudinal and interventional data, and inadequate integration of multi-omics approaches. Overall, available evidence suggests that gut dysbiosis is not simply a consequence of renal impairment, but a mechanistically relevant and potentially modifiable mediator of endocrine and metabolic complications in CKD. A better understanding of microbiota–metabolite–host interactions may support precision nutrition and microbiota-targeted therapies for risk stratification and treatment in renal endocrinology.

## 1 Introduction

Chronic kidney disease (CKD) has emerged as a major global public health challenge, affecting approximately 10% of the world’s population (1). Beyond the progressive decline in renal function, patients with CKD frequently develop a constellation of complex endocrine and metabolic derangements, including disorders of mineral and bone metabolism, insulin resistance, protein–energy wasting, and anaemia, that substantially elevate cardiovascular risk and all-cause mortality (2). In recent years, the gut microbiota and its metabolic products have attracted considerable attention for their role in CKD pathophysiology, giving rise to the central concept of the “gut–kidney axis” (3). Under CKD conditions, both the composition and functional capacity of the gut microbiota undergo marked alterations, a state referred to as gut dysbiosis, characterized by an expansion of bacteria that generate uremic toxins and a concomitant decline in species that produce beneficial metabolites (4, 5). This imbalance arises not only from the accumulation of metabolic waste products consequent to impaired renal excretory function, but also from the effects of medications commonly prescribed in CKD, dietary restrictions (such as low-fibre and fluid-restricted diets), and alterations in gut motility (6). The aberrant metabolites produced by a dysbiotic microbiota, together with depletion of protective metabolites, exacerbate systemic inflammation, oxidative stress, and endocrine–metabolic dysregulation through multiple mechanistic pathways, thereby establishing a vicious cycle that accelerates CKD progression and its attendant complications (7, 8).

**Figure 1:**
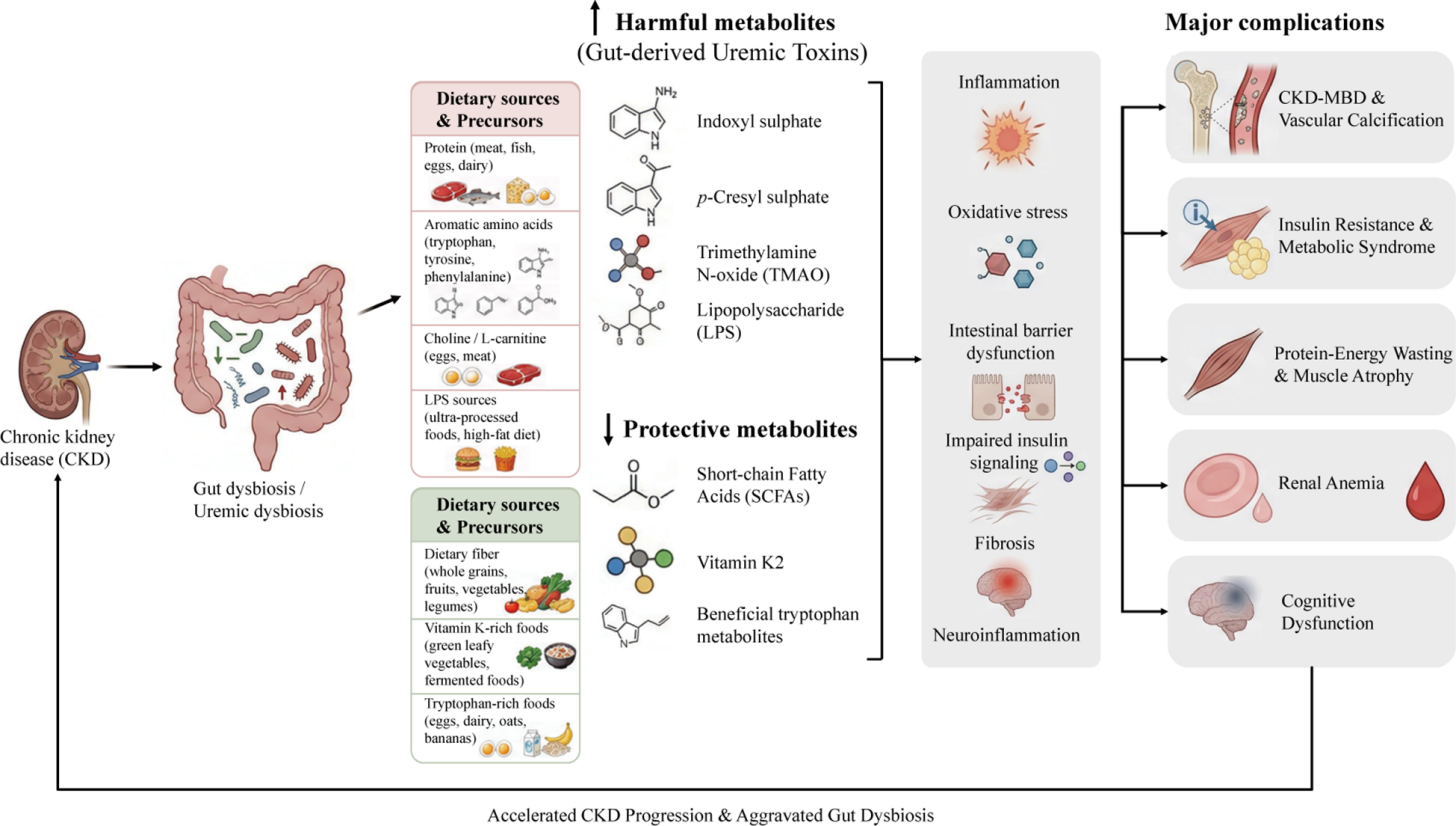
Schematic overview of the role of gut dysbiosis in endocrine and metabolic derangements of chronic kidney disease. Gut dysbiosis in CKD increases harmful gut-derived uremic toxins and decreases protective microbial metabolites, thereby inducing inflammation, oxidative stress, intestinal barrier dysfunction, impaired insulin signalling, fibrosis, and neuroinflammation. These changes contribute to CKD-mineral and bone disorder, vascular calcification, insulin resistance, protein–energy wasting, renal anaemia, cognitive dysfunction, and progressive worsening of CKD.

This mini-review, therefore, aims to provide an in-depth appraisal of the mechanisms by which gut dysbiosis drives endocrine and metabolic derangements in CKD, to critically examine the prevailing academic controversies, to identify key research gaps, and to explore future directions for therapeutic intervention, considerations of paramount clinical importance for improving outcomes in patients with CKD (**Figure 1**).

## 2 The gut microbiota and chronic kidney disease: an overview of the gut–kidney axis and the central role of endocrine–metabolic derangements

Although widely used, “dysbiosis” lacks an accepted operational definition: an altered microbiome in one disease context may be neutral, or even health-associated, in another (9). We therefore use the term here in a CKD-specific, functional sense: an expansion of proteolytic, uremic-toxin−generating taxa together with depletion of fibre-fermenting, SCFA-producing commensals, accompanied by reduced microbial gene richness. A bidirectional and intimate relationship exists between CKD and gut dysbiosis, forming the complex “gut–kidney axis” (3, 10). As CKD progresses, declining renal excretory capacity leads to the accumulation of nitrogenous waste products, including urea, in the systemic circulation; these diffuse across the intestinal wall into the gut lumen. The resultant elevation in intraluminal urea concentrations alters the local milieu, favouring the overgrowth of proteolytic bacteria (such as Enterobacteriaceae and Clostridium spp.) at the expense of saccharolytic commensals (notably short-chain fatty acid–producing species), thereby precipitating intestinal dysbiosis (4, 11). This condition has been termed “uremic dysbiosis” (5). Furthermore, dietary modifications commonly imposed on CKD patients, including potassium- and phosphate-restricted diets that curtail fruit and vegetable intake, as well as fluid restriction, reduced physical activity, and the frequent administration of phosphate binders, iron supplements, and antibiotics, all serve to compound the disruption of the intestinal microbiome (12, 13).

The gut is not merely an excretory organ; it functions as an active endocrine and metabolic organ. A healthy gut microbiota, through its metabolic activity, generates a diverse array of bioactive small molecules that enter the systemic circulation and remotely modulate the function of distant organs, a process consistent with the framework of “remote sensing and signalling” (7, 14). In the context of CKD, the cardinal consequence of gut dysbiosis is a profound alteration of the metabolite profile: on the one hand, the production and absorption of protein-bound uremic toxins (such as indoxyl sulphate and p-cresyl sulphate) and gut-derived toxins (such as trimethylamine N-oxide [TMAO]) are increased; on the other hand, metabolites with renoprotective properties (such as short-chain fatty acids [SCFAs] and certain tryptophan derivatives) are produced in insufficient quantities (15, 16). These alterations directly or indirectly disrupt host metabolic and endocrine homeostasis.

Endocrine and metabolic derangements occupy a central position in the spectrum of CKD complications. Mineral and bone disorder (CKD-MBD), for instance, is directly linked to vascular calcification and fracture risk; insulin resistance and the metabolic syndrome are important drivers of cardiovascular events in CKD; protein–energy wasting and muscle atrophy profoundly impair quality of life and survival; and renal anaemia and cognitive dysfunction are closely intertwined with metabolic abnormalities (1, 2, 17). A growing body of evidence indicates that the gut microbiota and its metabolites serve as a critical nexus linking CKD to these endocrine–metabolic complications (18, 19). Elucidating how gut dysbiosis drives these derangements through its metabolite network is therefore essential both for understanding the systemic consequences of CKD and for identifying novel therapeutic targets.

## 3 Core mechanisms by which gut dysbiosis drives endocrine–metabolic derangements in CKD: metabolite imbalance

The impact of gut dysbiosis on the endocrine–metabolic system in CKD is mediated principally through an imbalance of microbial metabolites. This imbalance manifests as the excessive accumulation of deleterious metabolites coupled with a relative deficit of beneficial ones, together creating a toxic milieu that promotes disease progression.

### 3.1 Accumulation of gut-derived uremic toxins: the pathogenic roles of indoxyl sulphate, p-cresyl sulphate, and TMAO

Indoxyl sulphate (IS) and p-cresyl sulphate (PCS) are among the most extensively studied protein-bound uremic toxins, generated by bacterial metabolism of tryptophan and tyrosine, respectively (4) (dietary precursors: animal and plant protein; converted in the colon by tryptophanase−expressing *E. coli*/*Bacteroides* spp. and proteolytic *Clostridium* clusters, then hepatically sulphated). These toxins accumulate markedly in patients with CKD and promote endocrine–metabolic derangements through several mechanisms. IS and PCS induce oxidative stress and a state of chronic inflammation by activating pro-inflammatory pathways such as nuclear factor κB (NF-κB), thereby impairing insulin signalling and fostering insulin resistance (20, 21). They also stimulate renal tubular epithelial cells, vascular smooth muscle cells, and osteoblasts to release profibrotic and proclitic factors, directly contributing to CKD-MBD and vascular calcification (20, 22). Mechanistic evidence for IS-induced macrophage dysfunction and uremic atherosclerosis derives mainly from *in vitro*/rodent models (21), whereas the association between circulating IS/PCS and CKD progression and cardiovascular mortality is supported by human prospective cohort and meta-analytic studies (23).

Trimethylamine N-oxide (TMAO) is another gut-derived metabolite of interest. Its precursors are dietary choline, phosphatidylcholine, L-carnitine, and betaine (red meat, eggs, full-fat dairy, certain fish), metabolized by intestinal bacteria to TMA and oxidized by hepatic FMO3 to TMAO (24). In CKD, TMAO is markedly elevated owing to reduced renal clearance and dysbiosis (25), and augments vascular inflammation, atherosclerosis, and vascular calcification via ER stress, mitochondrial ROS, and glycolytic reprogramming (26). TMAO also perturbs bone metabolism (27). (rodent CKD models; not yet validated in humans). Whether TMAO is purely toxic or context-dependent remains debated (28).

### 3.2 Depletion of protective metabolites: reductions in short-chain fatty acids, vitamin K, and beneficial tryptophan metabolites

Short-chain fatty acids (SCFAs; butyrate, propionate, acetate) are produced by bacterial fermentation of dietary fibre (precursors: resistant starch, inulin, pectin and β−glucan, fermented by *Faecalibacterium prausnitzii*, *Roseburia* spp., *Eubacterium rectale* and *Bifidobacterium* spp.). SCFAs exert anti-inflammatory effects, maintain intestinal barrier integrity, modulate immunity, and enhance insulin sensitivity (16). Low-fibre diets and dysbiosis in CKD reduce SCFA production (29, 30), compromising barrier function and exacerbating endotoxemia. Butyrate supplementation upregulates renal organic anion transporter OAT1, facilitating uremic toxin secretion (30) (kidney proximal-tubule cell model; not yet replicated in patients).

Gut bacteria also synthesise vitamin K2 (menaquinones) (produced by *Bacteroides*, *Prevotella*, *Escherichia*, and *Eubacterium*; dietary sources include natto and aged cheeses), an essential cofactor for matrix Gla protein. CKD patients frequently exhibit vitamin K deficiency, accelerating vascular calcification (19, 31). Tryptophan can also be metabolised via alternative pathways to anti-inflammatory and neuroprotective derivatives (indole-3-aldehyde, kynurenic acid); in CKD, tryptophan metabolism is skewed towards the deleterious indole pathway (32, 33). *Lactobacillus johnsonii* produces cyclo(Pro-Trp), which promotes intestinal Ca²^+^ absorption and alleviates secondary hyperparathyroidism in CKD (34) (preclinical rat CKD-SHPT model).

### 3.3 Stickland-fermentation indole derivatives: IAA, IPA, ILA, and related metabolites

Beyond the indole/IS axis, structurally related tryptophan derivatives, indole-3-acetic acid (IAA), indole-3-propionic acid (IPA), indole-3-lactic acid (ILA), and the less well-characterised indole-3-butyric acid (IBA), are generated through Stickland fermentation. The principal producers are strict anaerobes such as Clostridium sporogenes and Peptostreptococcus anaerobius, with selected Bifidobacterium and Lactobacillus spp. Additionally, generating ILA via aromatic lactate dehydrogenase (35). IPA, the most consistently characterised member, signals through AhR, PXR, and TLR4 to strengthen the gut barrier and exerts antioxidant, anti-atherosclerotic, and neuroprotective effects. Critically, fermentable dietary fibre redirects microbial tryptophan flux from tnaA-mediated indole production towards beneficial Stickland products: fibre-degrading Bacteroides thetaiotaomicron cross-feeds monosaccharides that repress tnaA in indole producers (35). Current CKD findings on IAA are inconsistent, reflecting heterogeneous dietary fibre intake and the dual AhR-mediated effects of these ligands at uremic concentrations, a key methodological caveat for future studies.

## 4 Gut dysbiosis and CKD-specific endocrine–metabolic complications

The imbalance of gut microbial metabolites, acting through distinct molecular pathways, is deeply implicated in a range of endocrine–metabolic complications specific to CKD.

### 4.1 Mineral and bone metabolism disorder and vascular calcification

CKD-MBD and vascular calcification (VC) are leading causes of cardiovascular death in patients with CKD. Gut dysbiosis plays a pivotal role in these processes. Beyond the direct capacity of IS, PCS, and TMAO to stimulate the osteogenic trans differentiation of vascular smooth muscle cells, microbiota-derived lipopolysaccharide (LPS) is an important mediator. Prevotella copri, for instance, is enriched in both CKD animal models and patients, with its abundance positively correlated with aortic calcification scores; the LPS it produces accelerates high-phosphate–induced vascular calcification through activation of the Toll-like receptor 4 (TLR4)/NF-κB/NLRP3 inflammasome pathway (20). Dysbiosis additionally modulates the calcification process indirectly by affecting vitamin K metabolism and SCFA production (19, 31). With respect to bone metabolism, the gut microbiota and its metabolites (notably butyrate) regulate the differentiation and function of immune cells, including regulatory T cells and T-helper 17 cells, that are critical mediators of parathyroid hormone (PTH)–driven bone formation and resorption, suggesting that the microbiota may influence the phenomenon of “skeletal resistance” (36).

### 4.2 Insulin resistance and the metabolic syndrome

The metabolic syndrome, encompassing obesity, hyperglycaemia, dyslipidaemia, and hypertension, is both a major risk factor for and a common complication of CKD (37). Gut dysbiosis promotes insulin resistance through several mechanisms. Oxidative stress and chronic inflammation induced by deleterious uremic toxins directly impair insulin signalling pathways (21). The reduction in SCFAs resulting from dysbiosis attenuates their beneficial effects on insulin sensitivity and on the secretion of glucagon-like peptide-1 (GLP-1) (33). In addition, endotoxemia secondary to increased intestinal permeability triggers low-grade systemic inflammation, a core mechanism underlying insulin resistance (16). In obesity-associated CKD, high-fat diets can further alter the gut microbiota, augmenting the production of toxins such as TMAO and establishing a self-reinforcing cycle of adipose tissue inflammation, insulin resistance, and renal injury (38, 39).

### 4.3 Protein–energy wasting and muscle atrophy

Protein–energy wasting (PEW) and muscle atrophy are highly prevalent in patients with CKD and are strongly associated with adverse outcomes (2). Gut dysbiosis contributes to muscle wasting through multiple pathways. First, a chronic inflammatory state, driven by uremic toxins and endotoxemia, activates the ubiquitin–proteasome system and the myostatin signalling pathway, leading to increased muscle protein catabolism (17). Second, the secretion of appetite-regulating hormones (such as GLP-1 and cholecystokinin) is modulated by the gut microbiota and its metabolites; dysbiosis may induce anorexia, thereby reducing nutritional intake (13). Third, SCFA deficiency may impair muscle energy metabolism and anabolic signalling (40). Mitochondrial dysfunction and oxidative stress represent additional mechanisms by which dysbiosis promotes muscle atrophy (17). Clinical studies have observed correlations between gut microbiota composition and indices of muscle mass and function, lending support to the existence of a “gut–muscle axis” (40).

### 4.4 Anaemia and cognitive dysfunction

The principal cause of renal anaemia is erythropoietin deficiency, although inflammation and disordered iron metabolism are also contributory. Gut dysbiosis, by increasing the production of pro-inflammatory cytokines and certain uremic toxins (such as IS), may suppress erythropoiesis and perturb hepcidin metabolism, thereby exacerbating anaemia (12). Oral iron supplements used to treat CKD-associated anaemia may further aggravate intestinal dysbiosis, establishing yet another vicious cycle (12).

Cognitive dysfunction is another frequent complication of CKD. The gut microbiota influences brain function through the “gut–brain axis.” Deleterious metabolites generated by dysbiosis, including TMAO, IS, and PCS, may impair cognitive function by disrupting the blood–brain barrier, inducing neuroinflammation, and promoting oxidative stress (33, 41). Concurrently, diminished production of protective metabolites (such as SCFAs and beneficial tryptophan derivatives) attenuates their neuroprotective and neurotrophic effects (33, 42). Evidence indicates that alterations in the gut microbiota of patients with CKD are associated with cognitive decline, and microbiota-targeted interventions may represent a novel strategy for cognitive improvement (42).

## 5 Academic controversies and divergent perspectives

Although the role of the gut microbiota in CKD is broadly acknowledged, several specific issues remain the subject of academic debate and divergent perspectives.

### 5.1 The TMAO debate: toxic biomarker versus potential physiological regulator

The prevailing body of evidence characterizes TMAO as a deleterious agent that promotes atherosclerosis, vascular calcification, and renal fibrosis (22, 24, 26). However, an alternative view holds that the effects of TMAO may be tissue-specific and context-dependent. In certain studies of metabolic disease, TMAO has exhibited bidirectional effects on insulin secretion and complex influences on energy metabolism, suggesting that it may function not merely as a toxic metabolic end-product but as a regulatory molecule whose role varies with the prevailing physiological or pathological milieu (28). Moreover, fish, a natural dietary source of TMAO, is also rich in omega-3 fatty acids. Whether the cardiovascular benefits conferred by fish consumption offset the potential risks associated with its TMAO content remains unresolved, prompting debate regarding whether CKD patients should increase their fish intake (43). Thus, whether TMAO represents a modifiable pathogenic factor or simply a concomitant biomarker reflecting gut dysbiosis and declining renal function requires further clarification through prospective interventional studies.

### 5.2 The risks and benefits of low-protein diets: retarding progression versus the risk of malnutrition

A low-protein diet (LPD) is a traditional nutritional strategy for retarding the progression of non-dialysis CKD, founded in part on the rationale of reducing the generation of nitrogenous waste products and uremic toxin precursors (44, 45). Studies have demonstrated that high-protein diets increase the generation of toxins such as IS and upregulate renal transporter expression, adversely affecting tubular function (14). Conversely, excessively stringent protein restriction, particularly in elderly patients or those already predisposed to wasting, may precipitate or exacerbate PEW and muscle atrophy, paradoxically increasing mortality risk (2, 44). The optimal scope of LPD implementation, the degree of protein restriction, and the means of individualizing the balance between renal protection and nutritional maintenance thus remain contentious issues in clinical practice. Current consensus favours an individualized, moderate protein restriction implemented under rigorous nutritional surveillance (44).

### 5.3 Dietary fibre and dietary patterns: microbial benefit versus electrolyte risk

A related controversy concerns dietary fibre and dietary patterns. Higher fibre intake consistently reduces serum IS/PCS and inflammation in CKD, with the mechanism now elucidated: fibre-derived monosaccharides repress *tnaA* in indole producers and redirect tryptophan towards beneficial Stickland products (35). However, fibre-rich plant foods often co-segregate with high potassium and phosphate, raising concerns about hyperkalaemia and hyperphosphataemia in stage 4–5 disease. Recent KDOQI/KDIGO updates and prospective cohorts suggest that plant-dominant patterns can be safely implemented at all CKD stages with individualised electrolyte monitoring, although no whole-diet pattern (Mediterranean, plant-dominant low-protein, DASH) has yet been shown by large randomised trials to improve hard end-points in advanced CKD.

## 6 Current research gaps and challenges

### 6.1 Insufficient depth of mechanistic investigation: establishing causality and integrating signalling pathways

The majority of studies to date have identified associations between gut dysbiosis and CKD complications, but definitive causal relationships have not been fully established. For instance, whether specific alterations in the microbiota cause vascular calcification, or whether the systemic milieu associated with calcification reciprocally shapes a particular microbiota, remains unclear (19). Moreover, although the signalling pathways through which various microbial metabolites (such as TMAO, IS, and SCFAs) exert their effects, including the NF-κB, aryl hydrocarbon receptor (AhR), and nuclear factor erythroid 2–related factor 2 (Nrf2) pathways, have been partially delineated, how these pathways interact to form a network and exert synergistic or antagonistic effects across different organs (gut, liver, kidney, vasculature, bone, and brain) remains to be elucidated through systematic, integrative research (32, 46). Understanding of the dynamic changes in the gut microbiota during the transition from acute kidney injury to CKD, and the mechanisms underlying these changes, is also relatively limited (47).

### 6.2 Methodological limitations: integration and interpretation of multi-omics data and the paucity of longitudinal studies

This field relies heavily on high-throughput technologies such as metagenomics and metabolomics. Although these technologies generate vast quantities of data, the effective integration of metagenomic, metabolomic, proteomic, and transcriptomic datasets and their precise correlation with clinical phenotypes remains a formidable methodological challenge (11, 48). Most clinical studies have employed cross-sectional designs, which can furnish only associative evidence; longitudinal studies that track dynamic changes in the microbiota and its metabolites during CKD progression, as well as long-term outcomes following intervention, are lacking (42, 48). Moreover, existing research has focused predominantly on bacteria, whereas the roles of the gut mycobiome (fungal communities), archaea, and virome in CKD remain largely unexplored, representing an area of “dark matter” awaiting investigation (49).

## 7 Diet-related therapeutic approaches

To complement these mechanistic insights, several diet-related strategies act through modulation of the microbiota. First, individualised moderate protein restriction (0.6–0.8 g/kg/day in non-dialysis CKD), preferential substitution of animal by plant protein, and ketoanalogue supplementation reduce the precursor flux to IS, PCS, and TMA while preserving lean body mass (44, 45). Second, increased fermentable fibre intake (target ≥ 25–30 g/day) lowers serum IS/PCS and redirects tryptophan metabolism towards beneficial Stickland products such as IPA (29, 35). Third, whole-diet patterns including the Mediterranean diet and plant-dominant low-protein diet (PLADO) integrate these principles and show favourable effects on uremic toxin burden and inflammation (49, 50). Fourth, targeted prebiotics, probiotics, synbiotics, postbiotics, polyphenol-rich nutraceuticals (e.g., curcumin), and menaquinone-rich fermented foods complement these patterns (29, 31, 34). Individualised electrolyte and phosphate monitoring is essential in advanced CKD, and large pragmatic randomised trials of integrated dietary patterns in stage 4–5 CKD remain a key unmet need.

## 8 Future directions

Future therapeutic approaches should move beyond the paradigm of “one probiotic for all.” By integrating metagenomic sequencing, metabolomic profiling, and machine learning algorithms, it may become possible to identify specific microbial or metabolite signatures associated with distinct CKD complications, such as rapid progression, severe vascular calcification, or refractory anaemia (51, 52). Machine learning has already been applied, for instance, to distinguish the metabolomic profiles of paediatric CKD according to aetiology (51). Building upon such advances, individualized nutritional plans (precision dietary fibre supplementation, personalized protein intake recommendations) or microbial preparations (tailored probiotic or synbiotic combinations) could be developed to achieve precision remodelling of the intestinal microecology (49, 50). The establishment of large-scale, phenotypically rich CKD patient microbiome and metabolome databases will be essential to underpin these efforts.

## 9 Conclusions

In summary, gut dysbiosis, through imbalance of its metabolic products, accumulation of deleterious uremic toxins and depletion of protective metabolites, plays a central driving role in the pathogenesis and progression of endocrine–metabolic derangements in CKD. It is deeply implicated in the pathophysiology of mineral and bone disorder, vascular calcification, insulin resistance, muscle atrophy, anaemia, and cognitive dysfunction, constituting a complex gut–kidney–bone–vasculature–brain axis. Although academic controversies persist regarding the role of TMAO, the operational definition of dysbiosis, the optimal use of dietary fibre and whole-diet patterns, the optimisation of low-protein diets, and the generalisability of probiotic interventions, targeting the gut microbiota, in particular through integrated, fibre-rich, plant-dominant dietary approaches, opens a promising avenue for the management of CKD and its complications.
